# Remarks on the Urinometer

**Published:** 1847-01

**Authors:** W. S. W. Ruschenberger

**Affiliations:** U. S. N.


### MEDICAL EXAMINER

AND

### RECORD OF MEDICAL SCIENCE.

NEW SERIES.—No. XXV.—JANUARY, 1847.

### ORIGINAL COMMUNICATIONS.

*Remarks on the Urinometer.* By W. S. W. RUSCHENBERGER, M. D., U. S. N.

U. S. NAVAL HOSPITAL,
*New York, December 5th,* 1846.

MY DEAR SIR,—Those members of the profession who may be engaged in investigating the changes induced in the constitution of urine by disease, may be led into error by using a gravimeter or urinometer, manufactured in Philadelphia, and known as Prout's, for ascertaining the specific gravity of the fluid. The instrument to which I allude consists of a copper ball, surmounted by a graduated stem of box-wood, balanced below by a brass wire and pea. The point at which the stem floats in distilled water is marked at zero, and the point at which it stands in a saturated solution of common salt, forms the opposite extremity of the scale, the interval being divided into sixty degrees.

If placed gently in a fluid under examination it will stand, say for illustration at 30°, but immerse the instrument entirely, so as to completely wet the stem, it will rise and fall, and become quiescent at 20°. I have repeated the experiment very frequently with the same result, in the same fluid, after wiping the stem dry, and in different fluids. I have also compared the specific gravity of fluids ascertained by this gravimeter, with the specific gravity of the same fluids ascertained by an accurately made specific gravity bottle, and found the result different in every instance, and without any corresponding ratio of difference.

Supposing it possible that the error might be confined to the particular instrument in my possession, I submitted it to the manufacturer for adjustment, and took the opportunity to compare it with four other instruments then on sale in his shop. We found that no two of the five instruments corresponded in their respective indications of specific gravity; and further, that every instrument varied from 6 to 10 degrees in its indication of specific gravity, according as it was gently permitted to subside into the fluid, or entirely immersed so as to wet the whole stem or scale. The price of the instrument with the case is seven dollars; its value for practical purposes is not a cent. The indications by it are too uncertain and too irregular to be even proximative to accuracy.

This instrument was exhibited at the fair of the Franklin Institute in 1845, but whether it received approval or not I do not remember. The objections to it might possibly be removed, if, instead of box-wood, metal were used for the gratuated scale or stem of the instrument.

The hydrometers of Beaumé and Gay Lussac are almost equally fallacious. After very numerous experiments the conclusion arrived at is, that the only true way of arriving at the specific gravity of urine or other fluid, is by means of the thousand-grain bottle and counterpoise.

If you consider the above information of any interest, it may be worth a place in the "Medical Examiner."

Very respectfully,
W. S. W. Ruschenberger.

Professor R. M. Huston, *Philadelphia.*

